# Coronary function testing vs angiography alone to guide treatment of angina with non-obstructive coronary arteries: the ILIAS ANOCA trial

**DOI:** 10.1093/eurheartj/ehaf580

**Published:** 2025-08-12

**Authors:** Coen K M Boerhout, Hanae F Namba, Tommy Liu, Marcel A M Beijk, Peter Damman, Martijn Meuwissen, Peter Ong, Udo Sechtem, Yolande Appelman, Colin Berry, Javier Escaned, Amir Lerman, Timothy D Henry, Pim van der Harst, Ronak Delewi, Jan J Piek, Tim P van de Hoef

**Affiliations:** Department of Cardiology, Amsterdam University Medical Center, Location AMC, Amsterdam, The Netherlands; Department of Cardiology, Amsterdam University Medical Center, Location AMC, Amsterdam, The Netherlands; Department of Cardiology, University Medical Center Utrecht, Heidelberglaan 100, 3584 CX Utrecht, The Netherlands; HartKliniek Rijswijk, Rijswijk, The Netherlands; HartKliniek Bilthoven, Bilthoven, The Netherlands; Department of Cardiology, Amsterdam University Medical Center, Location AMC, Amsterdam, The Netherlands; Department of Cardiology, Radboud University Medical Center, Nijmegen, The Netherlands; Department of Cardiology, Amphia Hospital, Breda, The Netherlands; Department of Cardiology and Angiology, Robert Bosch Krankenhaus, Stuttgart, Germany; Department of Cardiology and Angiology, Robert Bosch Krankenhaus, Stuttgart, Germany; Department of Cardiology, Amsterdam University Medical Center, Location VUMC, Amsterdam, The Netherlands; British Heart Foundation Glasgow Cardiovascular Research Centre, University of Glasgow, Glasgow, UK; Department of Cardiology, Golden Jubilee National Hospital, Clydebank, UK; Cardiology Department, Hospital Clínico San Carlos, IDISSC Universidad Complutense de Madrid, Madrid, Spain; Department of Cardiovascular Medicine, Mayo Clinic, Rochester, MN, USA; The Carl and Edyth Lindner Research Center at the Christ Hospital, Cincinnati, OH, USA; Department of Cardiology, University Medical Center Utrecht, Heidelberglaan 100, 3584 CX Utrecht, The Netherlands; Department of Cardiology, Amsterdam University Medical Center, Location AMC, Amsterdam, The Netherlands; Department of Cardiology, Amsterdam University Medical Center, Location AMC, Amsterdam, The Netherlands; Department of Cardiology, University Medical Center Utrecht, Heidelberglaan 100, 3584 CX Utrecht, The Netherlands

**Keywords:** Angina with non-obstructive coronary arteries, Coronary function testing, Coronary microvascular dysfunction, Coronary vasospasm

## Abstract

**Background and Aims:**

Invasive coronary function testing (CFT) identifies coronary vasomotor disorders in up to 90% of patients with angina with non-obstructive coronary arteries (ANOCA). The ILIAS ANOCA trial hypothesized that routine ad hoc CFT would be feasible, safe, and effective in providing an early, comprehensive diagnosis. Additionally, it was anticipated that combining CFT with a disease-specific treatment protocol would significantly improve quality of life in ANOCA patients compared with standard care.

**Methods:**

After excluding patients with obstructive coronary artery disease (CAD) during clinically indicated invasive coronary angiography (ICA), eligible patients underwent CFT and were randomized to either the standard care group, where CFT results remained blinded, or the intervention group, where CFT results were disclosed along with a tailored medical therapy protocol. The primary outcome was the mean difference in the within-subject change in Seattle Angina Questionnaire summary score (SAQSS) between groups from baseline to a follow-up of 6 months. The trial is registered with the International Clinical Trials Registry Platform (NL-OMON20739).

**Results:**

A total of 255 patients consented, of whom 153 patients (60%) without CAD underwent CFT and were randomized 1:1 to the standard care (*n* = 76) or intervention group (*n* = 77). All CFT procedures were successful without adverse events. A vasomotor disorder was identified in 120 patients (78%). At 6-month follow-up, the SAQSS improved significantly in the intervention group compared with the control group, with an intervention effect of 9.4 units (95% confidence interval 3.9–14.9, *P* = .001). There were no major adverse cardiac events at the 6-month follow-up.

**Conclusions:**

Routine CFT during the initial ICA was feasible, safe, and had high diagnostic yield. Implementing a pragmatic CFT protocol combined with a disease-specific treatment protocol significantly improved disease-related quality of life in patients with ANOCA compared with standard care.

## Structured Graphical Abstract

**Structured graphical abstract:**
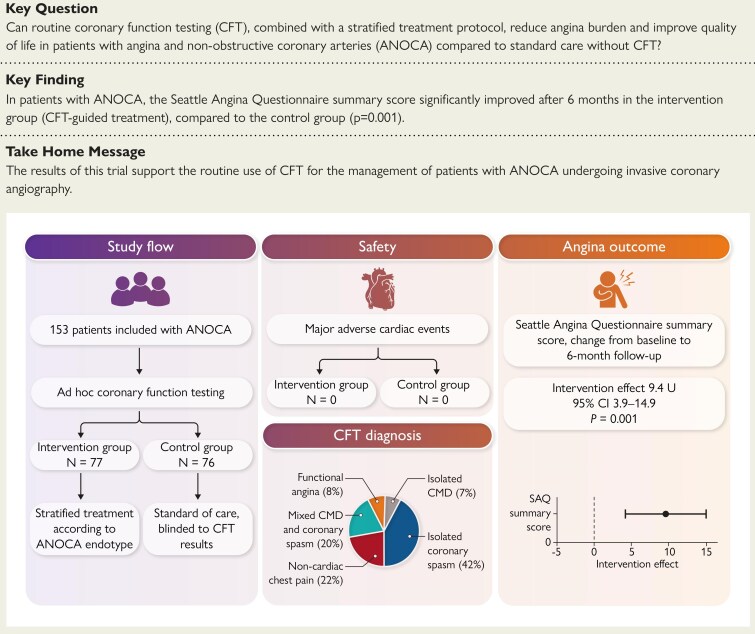
Graphical summary of the study flow, safety outcomes, CFT diagnoses, and primary outcome of Seattle Angina Questionnaire Summary Score change from baseline to a follow-up of 6 months. ANOCA, angina with non-obstructive coronary arteries; CFT, coronary function testing; CI, confidence interval; CMD, coronary microvascular dysfunction; SAQ, Seattle Angina Questionnaire

**See the editorial comment for this article ‘Angina with non-obstructive coronary arteries: a success story’, by R.A. Montone**  ***et al.*****, https://doi.org/10.1093/eurheartj/ehaf692.**

## Introduction

The diagnostic and therapeutic approach for patients with chronic coronary syndromes (CCS) is evolving from a simplistic approach focused on diagnosis and treatment of obstructive epicardial coronary artery disease (CAD) to include a comprehensive assessment of the functional status of the coronary circulation to assess causes of angina with non-obstructive coronary arteries (ANOCA).

ANOCA is present in 50%–70% of patients with CCS who are referred for invasive coronary angiography (ICA) and can originate from a diverse range of pathophysiological mechanisms.^1,2^ Invasive coronary function testing (CFT) involves additional assessment of coronary vasomotion and microvascular function to test for coronary vasomotor function disorders during ICA. Notably, when CFT is performed, underlying coronary vasomotor disorders are detected in up to 90% of patients.^3–5^ These disorders include impaired (microvascular) vasodilation and epicardial and/or microvascular vasospasm, reflecting functional and/or structural abnormalities of the coronary (micro)circulation.^6–8^ Each of these conditions has been associated with significant burden of anginal symptoms, impaired quality of life, increased risk of major adverse cardiovascular events, and substantial healthcare utilization and associated costs.^1,2^

The European Society of Cardiology has recently recommended CFT for persistently symptomatic patients with ANOCA despite medical treatment (Class I, Level of Evidence B) to identify potentially treatable ANOCA endotypes and to improve symptoms and quality of life. As such CFT is typically reserved for the situation where initial medical therapy has failed and involves a second invasive procedure.^2^ However, given that 50%–70% of patients undergoing ICA have ANOCA, implementing ad hoc CFT during the initial ICA could reduce time to diagnosis without the need for ancillary invasive procedures and may lead to earlier symptomatic improvement. Both the Coronary Microvascular Angina (CorMicA) and Advanced Invasive Diagnosis for Patients with Chronic Coronary Syndromes Undergoing Coronary ANGIOgraphy (AID-ANGIO) studies previously demonstrated a high diagnostic yield of ad hoc CFT in patients presented to the catheterization laboratory for suspected CAD.^4^ The CorMicA trial additionally concluded that an ad hoc CFT approach to guide medical therapy improves quality of life in ANOCA patients, but further evidence supporting the clinical benefit of this strategy remains limited.^9^

The ILIAS ANOCA (Inclusive Invasive Physiological Assessment in Angina Syndromes—Angina with No Obstructive Coronary Artery Disease) trial aimed to explore the clinical utility of ad hoc CFT in a multicentre setting. We hypothesized that a routine ad hoc CFT strategy, combined with a disease-specific treatment protocol, would lead to a significant improvement in quality of life among patients with ANOCA compared with standard care. Additionally, we proposed that routine ad hoc CFT would be both feasible and safe in routine clinical practice, offering an effective means of providing an early and comprehensive diagnosis.

## Methods

### Study design and patient population

The ILIAS ANOCA trial is an investigator-initiated prospective, randomized, blinded-arm controlled trial designed to compare standard care with the addition of CFT and tailored medical therapy in patients with ANOCA. The study design has been published previously.^10^ The study was conducted across five cardiac centres in the Netherlands (Amsterdam UMC, Amsterdam; Radboud UMC, Nijmegen; UMC Utrecht, Utrecht; and Amphia Hospital, Breda) and Germany (Robert Bosch Krankenhaus, Stuttgart). The design is similar to the CorMicA trial.^9^ The trial is registered with International Clinical Trials Registry Platform (NL-OMON20739).

Patients referred for clinically indicated ICA as standard of care in the diagnostic work-up for CCS were eligible for enrolment. Exclusion criteria were age younger than 18 years (no upper age limit), non-coronary indications for ICA (e.g. valvular heart disease), ICA performed in the evaluation of elevated cardiac troponin (myocardial infarction with no obstructed coronary arteries), significant renal impairment, obstructive epicardial CAD on ICA [defined as angiographical diameter stenosis >50%, a fractional flow reserve (FFR) ≤ .80, or a non-hyperaemic pressure ratio (NHPR) ≤ .89], or inability to consent. Eligible patients underwent CFT either ad hoc during the initial ICA or as a staged procedure within 4 weeks if immediate CFT was not feasible. The investigators were urged to pursue an ad hoc CFT approach and to stage CFT-only approach as a bailout strategy.

### Invasive coronary angiography and invasive coronary function testing protocol

ICA was performed according to standard clinical care at the participating hospitals. Patients continued all medications as per normal care. The use of radial cocktail was at the discretion of the interventional cardiologist. ICA was performed after intracoronary administration of nitroglycerine (200–300 µg) and could include FFR or NHPR assessments to exclude hemodynamically significant obstructive CAD at the discretion of the operator. Once obstructive CAD was ruled out, a standardized ad hoc CFT protocol was initiated.^10^ In short, a dual pressure and Doppler crystal-equipped guidewire (ComboWire, Philips, The Netherlands) or, in the absence of discernible CAD, a Doppler crystal-equipped guidewire (FloWire, Philips, The Netherlands) was introduced in the left anterior descending coronary artery (LAD) for assessment of coronary flow reserve (CFR) and hyperaemic microvascular resistance (HMR). If, at the discretion of the operator, anatomy precluded safe instrumentation of the LAD, the left circumflex coronary artery was instrumented instead. Hyperaemia was induced by an intracoronary bolus of 200 µg of adenosine, and measurements were performed in duplicate. The highest CFR value and corresponding HMR value were used to determine microvascular function, where CFR ≤2.5 and/or HMR ≥2.5 mmHg/cm/s were considered abnormal. Subsequently, and at least 10 min after the last nitroglycerine administration, acetylcholine (ACh) provocation testing was performed by manual intracoronary administration of ascending doses of 2, 20, 100, and 200 μg ACh over approximately 60 s. After each individual dose, care was taken to ensure that the effect of ACh had fully waned before proceeding with the next administration. This was confirmed by the return of flow velocity to baseline values. If significant vasospasm was identified at any step, no further escalating doses were administered. After the final dose of ACh, or after identification of significant coronary spasm, 200–300 µg of nitroglycerine was administered, followed by repeat coronary angiography. Finally, the highest ACh dose that induced coronary spasm during the initial test was re-administered as a re-challenge to evaluate co-existence of microvascular spasm.^11^ If ACh provocation testing was negative in the left coronary artery (LCA), the right coronary artery (RCA) was instrumented, and a single intracoronary dose of ACh (80 μg in 60 s) was administered.

### Randomization, implementation, and blinding

After completion of the CFT, participants were randomly assigned (1:1) to either the intervention group (where CFT results, including the tailored treatment protocol, were disclosed) or the standard care group (where CFT results remained undisclosed). Randomization followed a concealed, electronically generated sequence with variable block lengths (20 allocations consisting of four blocks: two of length four and two of length six, in random order). In the intervention group, CFT results were disclosed to both the patient and the referring cardiologist. In the standard care group, patients and referring cardiologists received only the ICA results and were advised to follow local clinical standards and to adhere to contemporary practice guidelines without access to CFT findings.

### ANOCA endotypes and medical therapy approach

In the ILIAS ANOCA trial, we defined five distinct ANOCA endotypes: coronary microvascular dysfunction (CMD), coronary vasospasm (whether epicardial or microvascular), a combination of both (mixed vasomotor disorders), functional angina, or non-cardiac chest pain. CMD was defined as the presence of either an impaired CFR (≤2.5) or an increased HMR (≥2.5 mmHg/cm/s). Epicardial coronary vasospasm was defined according to the COVADIS (Coronary Vasomotion Disorders International Study Group) criteria, which requires that three conditions are satisfied during ACh testing: (i) clinically significant epicardial vasoconstriction (≥90%), (ii) reproduction of the recognizable chest pain, and (iii) ischaemic ECG changes. Following COVADIS criteria, microvascular spasm was defined as (i) reproduction of the recognizable chest pain and (ii) ischaemic ECG changes, but in the absence of clinically significant epicardial vasoconstriction (<90%). Functional angina included those patients with reproduction of the usual chest pain during infusion of ACh, in the absence of clinically significant epicardial vasoconstriction and ischaemic ECG changes. A diagnosis of non-cardiac chest pain required a normal response to both adenosine and ACh.

All the endotypes were subsequently linked to a stepwise medical therapy protocol to facilitate uniform treatment (see Supplementary data online, *Figure S1*). In the intervention group, standardized letters were sent to the referring cardiologist including the results of the CFT and advice on stepwise optimization of medical therapy in line with the final diagnosis during CFT. Standard care for patients in the control arm consisted of guideline-directed medical therapy and antianginal therapies according to the preference of the referring cardiologist. The referring cardiologist had discretion over the final treatment decisions in both the standard care and intervention arm of the study.

### Questionnaires and follow-up

The Seattle Angina Questionnaire (SAQ) is a validated, patient-reported questionnaire designed to assess the impact of angina on quality of life.^12^ The SAQ quantifies patients’ physical limitations caused by angina, the frequency of and recent changes in their symptoms, their satisfaction with treatment, and the degree to which they perceive their disease to affect their quality of life. Each domain score ranges from 0 to 100, with higher scores indicating better health status. The SAQ summary score (SAQSS) is a composite measure derived from three domains (angina limitation, frequency, and quality of life) to provide quantification of overall disease burden of angina.

Health status was further serially assessed using EuroQol (EQ-5D-5L), a validated, patient-reported instrument for measuring generic health-related quality of life, whereby higher scores represent better quality of life.^13^ It includes five dimensions: mobility, self-care, usual activities, pain/discomfort, and anxiety/depression, each rated on five levels of severity. Responses generate a five-digit health profile that can be converted into a single summary index score using a national value set. A separate visual analogue scale (VAS) allows patients to rate their overall health from 0 (worst) to 100 (best).

### Outcomes

The primary outcome was the mean difference in the within-subject change in SAQSS between groups from baseline to 6-month follow-up. Three pre-specified secondary outcomes are presented in this manuscript: (i) diagnostic yield of ad hoc CFT in ANOCA, (ii) feasibility and safety of ad hoc CFT, and (iii) within-subject change across the individual domains of the SAQ, as well as the EQ-5D-5L.

### Statistical analysis

The sample size was calculated to detect a change in the primary outcome; the between-group difference in the change in SAQSS from baseline to 6 months. To detect a mean group difference of change in SAQSS of 9 ± 19 units (U), a sample size of 70 patients per group was calculated to provide 80% power.^9^ This calculation assumed a two-tailed 5% significance level. To account for attrition, we planned to randomize 150 patients.

The primary outcome was analysed by means of a mixed-effects linear regression model, including a random effect for patients, and fixed effects for time point (baseline or follow-up), randomized group, and their interaction. Adequacy of this model was checked with standard diagnostic checks, including the assessment of residual normality, homoscedasticity, and the presence of outliers. The baseline-adjusted intervention effect was estimated as the interaction term from this model. The same approach was used for analyses of the individual components of the SAQ and the EQ-5D-5L. The EQ-5D-5L score was converted to the index value via the Dutch and German value set from the EuroQol crosswalk calculator spreadsheet.

Data are reported as mean ± SD, median (25th, 75th percentile), or frequency (%), as appropriate. We performed two-tailed analyses and considered a *P*-value <.05 to be significant. All analyses were conducted with Stata version 14.1 (StataCorp, College Station, TX, USA). Analyses were done according to the intention-to-treat principle.

### Role of the funding source

The funders of the study had no role in study design, data collection, data analysis, data interpretation, or writing of the report.

## Results

**Figure 1:**
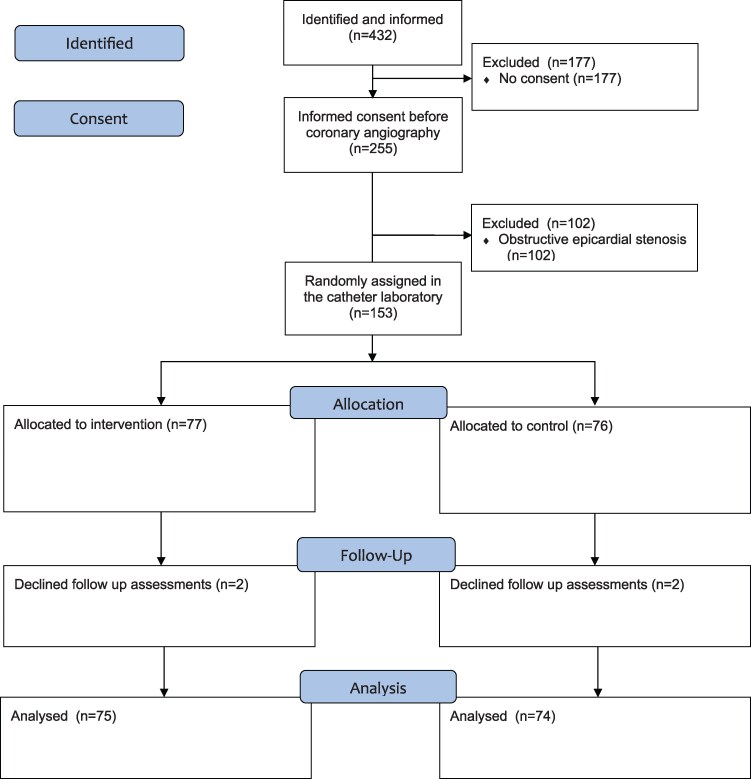
Trial flow diagram (CONSORT)

Between September 2021 and October 2024, a total of 432 patients were assessed for eligibility and met the study inclusion criteria. Of these, 255 consented and were subsequently evaluated during ICA. ICA revealed obstructive CAD in 102 patients (40%), based on either angiography or additional FFR/NHPR measurements. Ultimately, 153 patients were eligible for inclusion in the study and underwent CFT (*Figure 1*). Among these participants, 148 patients underwent direct ad hoc CFT during the initial ICA and 5 patients underwent staged CFT due to logistical constraints. In the patients where CFT was staged, the time to CFT was 3 ± 1 days from the initial ICA. All the patients undergoing CFT were randomized. Therefore, the final number of patients included in the intention-to-treat analysis was *n* = 77 in the intervention group and *n* = 76 in the standard care group.

### Baseline and procedural characteristics

*Table 1* summarizes the baseline characteristics of the final study population. The participants were predominantly women (*n* = 84, 55%) with a median age of 64 years (56–70 years). A total of 134 patients (88%) underwent non-invasive evaluation before ICA. Of these, 85 patients (56%) underwent non-invasive stress testing, which was positive for myocardial ischaemia in 32 patients (38%). Moreover, 49 patients (34%) underwent coronary computed tomography angiography (CCTA) prior to the ICA, which documented a Coronary Artery Disease Reporting and Data System (CAD-RADS) 3 or higher in 35 patients (71%).

**Table 1:** Baseline and clinical characteristics for the randomized study population and per randomization group

	All patients *n* = 153	CFT disclosed *n* = 77	CFT not disclosed *n* = 76
Patient characteristics			
Age (years)	64 (56,70)	65 (56,69)	64 (57,71)
Women, *n* (%)	84 (55)	41 (53)	43 (57)
BMI (kg/m^2^)	28.2 (24.3,30.9)	27.1 (24.9,29.0)	29.3 (24.4,31.9)
Hypertension, *n* (%)	74 (48)	40 (52)	34 (45)
Diabetes, *n* (%)	28 (18)	10 (13)	18 (24)
Dyslipidaemia, *n* (%)	79 (52)	41 (53)	38 (50)
Smoking, *n* (%)			
Current	14 (9)	8 (10)	6 (8)
Former	46 (30)	25 (32)	21 (28)
Family history of CVD	71 (46)	36 (47)	35 (46)
History of myocardial infarction	15 (10)	10 (13)	5 (7)
History of PCI	22 (14)	14 (18)	8 (11)
Women-specific risk factors			
History of early menopause	4 (5)	2 (5)	2 (5)
History of pregnancy-related hypertension	4 (5)	2 (5)	2 (5)
History of pre-eclampsia	2 (2)	1 (3)	1 (2)
Non-invasive diagnostics			
CT coronary angiography, *n* (%)	49 (32)	27 (35)	22 (29)
CAD-RADS 3 or higher, *n* (%)	35 (71)	17 (63)	18 (81)
Ischaemia testing, *n* (%)	85 (56)	44 (57)	41 (54)
Positive for ischaemia, *n* (%)	32 (38)	12 (27)	20 (49)
Seattle Angina Questionnaire			
Angina summary score	62 (48, 76)	59 (44, 75)	66 (53, 78)
Angina limitation	78 (58, 92)	72 (56, 92)	78 (64, 92)
Angina stability	50 (32, 74)	50 (28, 68)	50 (25, 75)
Angina frequency	70 (50, 90)	60 (40, 90)	80 (60, 90)
Angina treatment satisfaction	75 (56, 93)	75 (57, 99)	81 (63, 94)
Angina quality of life	42 (25, 58)	42 (25, 58)	50 (33, 67)
Quality of life (EQ-5-DL)			
Index score	0.75 (0.61, 0.86)	0.77 (0.61, 0.84)	0.75 (0.61, 0.89)
VAS score	69 (50, 79)	67 (51, 75)	70 (50, 80)

Values are median (Q1, Q3) or *n* (%).

CFT, coronary function testing; BMI, body mass index; CVD, cardiovascular disease; PCI, percutaneous coronary intervention; CT, computed tomography; CAD-RADS, Coronary Artery Disease Reporting and Data System; VAS, visual analogue scale.

At baseline, the median SAQSS across the whole study population was 62 (48–76), and amounted to 59 (44–75) in the intervention group and 66 (53–78) in the control group (*P* = .028). The EQ-5D-5L questionnaire showed a median index score of .75 (.61–.86) and a median VAS score of 69 (50–79).

*Table 2* shows the procedural characteristics. The LAD was studied with adenosine in 150 patients (98.0%), whereas the left circumflex coronary artery was studied in the other 3 patients (2.0%). The median FFR was .91 (.86–.94) and the median NHPR was .94 (.92–.97). Adenosine testing showed a median CFR of 2.9 (2.6–3.5), and a median HMR of 1.7 mmHg/cm/s (1.4–2.3 mmHg/cm/s).

**Table 2:** Procedural characteristics for the randomized study population, and per randomization group

	All patients *n* = 153	CFT disclosed *n* = 77	CFT not disclosed *n* = 76
Coronary angiography characteristics			
CFT procedure time (min)	21 (17, 25)	22 (17, 27)	21 (17, 25)
Stenosis present, *n* (%)	40 (26)	21 (27)	19 (25)
Stenosis diameter			
FFR	0.91 (0.86, 0.94)	0.92 (0.89, 0.97)	0.88 (0.86, 0.94)
NHPR	0.94 (0.92, 0.97)	0.93 (0.92, 0.95)	0.94 (0.92, 0.97
Adenosine testing			
Coronary flow reserve	2.9 (2.6, 3.5)	2.9 (2.6, 3.4)	2.9 (2.5, 3.5)
Hyperaemic microvascular resistance	1.7 (1.4, 2.3)	1.8 (1.4, 2.3)	1.7 (1.3, 2.3)
Acetylcholine testing			
Epicardial spasm present, *n* (%)	78 (51)	38 (49)	40 (52)
Diffuse, *n* (%)	64 (84)	32 (86)	32 (82)
Focal, *n* (%)	12 (16)	5 (14)	7 (18)
Microvascular spasm present, *n* (%)	33 (22)	18 (23)	15 (20)
ECG abnormalities, *n* (%)	100 (65)	52 (68)	48 (63)
Recognizable angina, *n* (%)	120 (78)	62 (80)	58 (76)
Final diagnosis			
Isolated CMD	11 (7)	5 (6)	6 (8)
Isolated coronary spasm	65 (42)	36 (47)	29 (38)
Combined CMD and coronary spasm	31 (20)	16 (21)	15 (20)
Functional angina	13 (8)	5 (6)	8 (10)
Non-cardiac angina	33 (22)	15 (20)	18 (24)

Values are median (Q1, Q3) or *n*(%). Isolated coronary spasm is defined by the presence of epicardial or microvascular coronary spasm according to the COVADIS criteria, functional angina is defined by the presence of recognizable angina in response to ACh without the presence of epicardial or microvascular spasm, and non-cardiac angina is defined by the absence of abnormalities according to the CFT.

CFT, coronary function testing; CMD, coronary microvascular dysfunction, defined by CFR < 2.5 or HMR > 2.5; ECG, electrocardiogram; FFR, fractional flow reserve; NHPR, non-hyperaemic pressure ratio.

Overall, a form of coronary vasomotor dysfunction was identified in 120 (78%) patients. CFT identified the following underlying ANOCA endotypes: isolated CMD in 11 patients (7%), isolated coronary vasospasm in 65 patients (42%), mixed CMD and coronary spasm in 31 patients (20%), functional angina in 13 patients (8%), and non-cardiac chest pain in 33 patients (22%). Of the patients with coronary vasospasm, 78 (81%) had epicardial spasm, of which 84% was diffuse and 16% was focal, and 33 (22%) had microvascular spasm. In the absence of discernible vasospasm in the LCA, the RCA was evaluated with ACh in 20 patients (13%). None (0%) of these patients had evidence of vasospasm in the RCA. In the other 13 patients (8%) with no evidence of vasospasm in the LCA, the RCA was considered unsuitable for ACh provocation by the operator.

### Safety and feasibility of ad hoc coronary function testing

CFT was successfully completed in 153 (100%) patients. The mean procedure time of the CFT protocol was 21 ± 6 min, including assessment of the RCA in 20 (13%) patients. During or directly after the CFT, no procedure-related complications were reported, and none of the CFT procedures were terminated preliminary. During the 6-month follow-up period, there were no major adverse cardiac events.

### Medication use

Medication use at baseline and 6-month follow-up is summarized in *Table 3*. At baseline, there were no significant differences in cardiac medication use between the intervention and standard care groups. At 6-month follow-up, a significantly higher proportion of patients in the intervention arm were treated with non-dihydropyridine and dihydropyridine calcium channel blockers (36% and 32%, respectively), compared with the standard care arm (14% and 13%, respectively; *P* < .005 for both). The use of short-acting nitrates was also significantly higher in the intervention group at follow-up (45% vs 25%; *P* = .008), driven by more frequent discontinuation in the standard care group. Beta-blocker use declined in both groups from baseline to follow-up, with no significant difference between arms at either timepoint. The use of aspirin, statins, angiotensin-converting enzyme inhibitors/angiotensin receptor blockers, long-acting nitrates, ranolazine, and ivabradine did not differ significantly between groups at follow-up.

**Table 3:** Medication use at baseline and 6-month follow-up

Medication	Baseline				6-month follow-up			
	All patients	CFT disclosed	CFT not disclosed	*P*-value^a^	All patients	CFT disclosed	CFT not disclosed	*P*-value^a^
Aspirin, *n* (%)	102 (67)	49 (64)	53 (70)	.42	78 (51)	40 (52)	38 (50)	.80
Statin, *n* (%)	114 (75)	54 (70)	60 (79)	.21	109 (71)	55 (71)	54 (71)	.95
ACEi or ARB, *n* (%)	39 (25)	20 (26)	19 (25)	.89	41 (27)	25 (32)	16 (21)	.11
Beta-blocker, *n* (%)	73 (48)	33 (43)	40 (53)	.22	39 (26)	17 (22)	22 (29)	.33
Non-DHP CCB, *n* (%)	24 (16)	14 (18)	10 (13)	.39	39 (25)	28 (36)	11 (14)	.002
DHP CCB, *n* (%)	28 (18)	15 (19)	13 (17)	.70	35 (23)	25 (32)	10 (13)	.004
Nitrates (SA), *n* (%)	68 (44)	39 (51)	29 (38)	.12	54 (35)	35 (45)	19 (25)	.008
Nitrates (LA), *n* (%)	20 (13)	11 (14)	9 (12)	.65	22 (14)	10 (13)	12 (16)	.62
Ranolazine, *n* (%)	1 (1)	0 (1)	1 (1)	.31	1 (1)	0 (1)	1 (1)	.31
Ivabradine, *n* (%)	27 (18)	18 (23)	9 (12)	.06	26 (17)	17 (22)	9 (12)	.09

Values are *n* (%).

ACEi, angiotensin-converting enzyme inhibitor; ARB, angiotensin receptor blocker; DHP, dihydropyridine; CCB, calcium channel blocker; SA, short-acting; LA, long-acting.

^a^ *P*-value derived from the difference in proportion across the two randomization groups.

### Impact on disease-related quality of life

**Figure 2:**
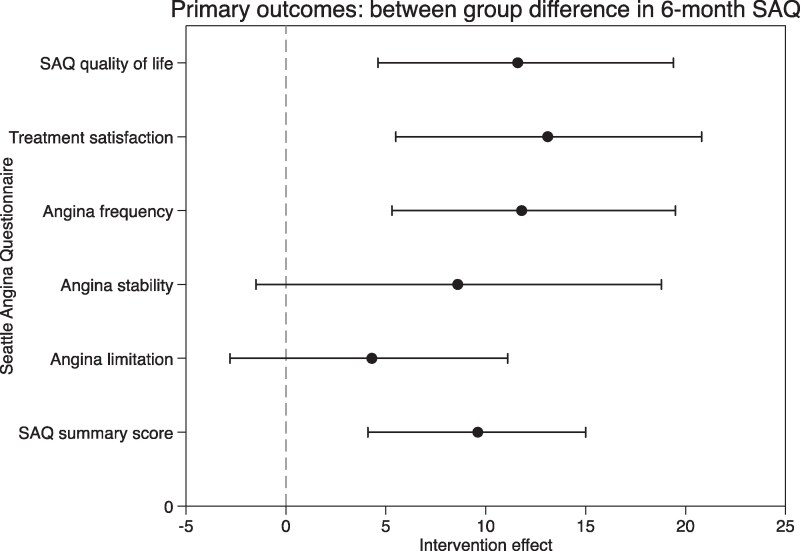
Between-group difference in change of SAQ component scores from baseline to 6 months. Forest plot of mean treatment difference in SAQ summary score (95% CI) and breakdown of the SAQ domains. The SAQ summary score is the mean of three angina domains (angina limitation, frequency, and quality of life). The angina summary score and the individual components were adjusted for baseline variation using a mixed linear regression model

Of the final study population, 149 patients completed the questionnaires at 6-month follow-up. The other four patients were lost to follow-up, of which two within the intervention group and two in the control group. The intervention resulted in a significantly greater improvement in SAQSS compared with standard care, with a mean difference of 9.4 U [95% confidence interval (CI) 3.9–14.9; *P* = .001] in favour of the intervention arm. This effect was primarily driven by a markedly greater improvement in angina frequency, with a difference of 11.6 U (95% CI 5.3–19.5; *P* = .001), and in SAQ-assessed quality of life, with a difference of 11.3 U (95% CI 4.6–19.4), both favouring the intervention arm. The difference in improvement in physical limitation, as a component of the SAQSS, did not reach statistical significance. In addition, treatment satisfaction improved significantly, with a difference of 12.9 U (95% CI 5.5–20.8; *P* = .001) in favour of the intervention arm. No significant intervention effect was observed for angina stability. The individual components of the SAQ domains are presented in *Table 4* and *Figure 2*. Diagnostic checks indicated that the model assumptions were adequately met.

**Table 4:** Primary outcome and changes in Seattle Angina Questionnaire at 6 months

	Control (*n* = 74)		Intervention (*n* = 75)		Intervention effect		
	6 months	Change from baseline	6 months	Change from baseline	Estimate	95% CI	*P*-value
Seattle Angina Questionnaire							
Angina summary score	71 (66 to 75)	5.3 (.11 to 12.5)	74 (69 to 78)	14.7 (10.8 to 18.6)	9.4	3.9 to 14.9	.001
Angina limitation	77 (71 to 83)	2.7 (−5.0 to 10.1)	79 (73 to 84)	6.9 (2.0 to 11.7)	4.2	−2.6 to 11.0	.226
Angina stability	53 (47 to 59)	3.0 (−5.0 to 10.8)	59 (53 to 65)	11.6 (4.3 to 18.6)	8.5	−1.5 to 18.5	.098
Angina frequency	76 (71 to 82)	4.7 (−.2 to 9.5)	79 (74 to 85)	16.5 (11.2 to 21.2)	11.6	5.3 to 19.5	.001
Treatment satisfaction	71 (66 to 76)	−4.9 (−10 to .4)	80 (75 to 85)	8.2 (2.7 to 13.4)	12.9	5.5 to 20.8	.001
SAQ quality of life	57 (52 to 63)	9.13 (3.9 to 14)	63 (57 to 68)	21.1 (15 to 26)	11.3	4.6 to 19.4	.001
EQ-5D-SL							
Index score	.75 (.69 to .79)	.02 (−.02 to .06)	.79 (.74 to .84)	.08 (.04 to .12)	.06	.005 to .12	.033
VAS score	70 (66 to 74)	5.3 (.5 to 8.3)	71 (67 to 75)	8.0 (3.4 to 11)	2.9	−2.7 to 8.4	.307

Values are derived from a mixed linear regression model and depicted as mean (95% CI). SAQ: lower scores represent worse angina symptom. VAS is a visual analogue score of EQ-5D validated quality of life too: higher scores indicate better quality of life.

Quality of life defined by the index score derived from the EQ-5D-5L questionnaire showed a significantly greater improvement in favour of the intervention arm, with a mean difference of .06 (95% CI .005–.12, *P* = .033). The EQ-5D-5L VAS score was not significantly impacted by the intervention.

## Discussion

The routine application of ad hoc CFT during clinically indicated ICA linked with a stepwise medical therapy protocol led to a significant improvement in angina burden and quality of life in patients with ANOCA. Ad hoc CFT was feasible and safe, without the occurrence of adverse events (see the Structured Graphical Abstract). The results of this randomized, controlled trial provide external validation of the CorMicA trial results^9^ and add support for contemporary guideline recommendations for use of CFT in patients with suspected ANOCA.^2,14^

Over a period of 3 years, we enrolled 432 outpatients referred for ICA to interventional cardiac centres across the Netherlands and Germany with expertise in CFT. Despite the challenges posed by the COVID-19 pandemic, followed by a year-long global shortage of ACh, we managed to include 153 patients of which 149 completed the 6-month follow-up. The implementation of an ad hoc CFT protocol was found safe and feasible with no complications and a mean additional procedure time of 21 ± 6 min. This is in contrast with previous reported incidences of complications by traditional CFT protocols and procedure times, which have contributed to the limited adoption in contemporary clinical practice.^15^ The most likely explanation for this difference is the use of manual bolus injection for the infusion of both ACh and adenosine, which allows for faster procedure times and direct control over injection speed to address potential ACh-related bradycardia. These safety and feasibility findings align with recent results from the NL-CFT registry, using a similar CFT protocol,^5^ and with results from the AID-ANGIO study.^4^

Importantly, the addition of ad hoc CFT to ICA resulted in a diagnostic yield of 87% compared with a diagnostic yield of 40% based on the ICA alone. Of these, most patients were diagnosed with coronary spasm, either alone (42%) or in combination with CMD (20%). Isolated CMD was present in 7%, functional angina in 8%, and non-cardiac chest pain in 22% of patients. These results align with previous studies using similar CFT protocols.^3,5^ Interestingly, the incidence of coronary vasospasm was similar compared with CFT protocols that withhold vasoactive medication 24–48 h prior to CFT and/or prohibit the use of radial cocktail and nitrates before ACh provocation testing is performed. These results support a pragmatic approach to CFT in routine clinical practice.

In the intervention arm of the study, disclosure of the results to both the patient and the referring cardiologist, along with provision of a stepwise treatment protocol, led to significant changes in medical therapy compared with the standard care arm. Consistent with the predominance of coronary vasospasm observed in the cohort, these changes were primarily characterized by increased use of calcium channel blockers and sustained high use of short-acting nitrates in the intervention arm. Additionally, in both groups, documentation of the absence of obstructive CAD frequently led to discontinuation of beta-blocker therapy, with no difference between groups at the 6-month follow-up. Thus, despite medical therapy being ultimately at the discretion of the treating cardiologist, the CFT-guided treatment protocol clearly influenced therapeutic decision-making.

Most importantly, the study showed that CFT-guided treatment in ANOCA using a stepwise medical therapy protocol led to a significant improvement in angina burden and patient-reported quality of life, highlighted by the clinically relevant improvement in SAQSS and EQ-5D-5L. Importantly, the magnitude of improvement in the SAQSS achieved by the intervention in the ILIAS ANOCA trial is substantially greater than that observed following revascularization in patients with CAD in the ISCHEMIA trial.^16^ Similarly, the effect of the intervention on the EQ-5D-5L index score exceeds the established minimal clinically important difference for patients with cardiovascular disease.^17^ These results are in line with the pioneering CorMicA trial, which reported a similar greater improvement following CFT-guided treatment in ANOCA compared with standard care (11.7 U in the SAQSS, and .10 in the EQ-5D-5L index score). Interestingly, the treatment effect of CFT-guided medical therapy in the ILIAS ANOCA trial was achieved in a less symptomatic patient population compared with the CorMicA trial, with a baseline mean SAQSS of 62 ± 19 in the ILIAS ANOCA trial vs 51 ± 18 in the CorMicA trial. Yet, patients in both ILIAS ANOCA and CorMicA trials were substantially more symptomatic than patients enrolled in the ISCHEMIA trial evaluating optimal treatment of CAD.^16^ The recent results from the WARRIOR trial highlight the importance of incorporating CFT to guide medical therapy in ANOCA, as it showed that intensive medical therapy without the guidance of CFT did not significantly reduce the first occurrence of major adverse cardiovascular events compared with usual care over 5 years in women with suspected ischaemia and non-obstructive coronary arteries (NCT03417388).

Overall, the findings in the ILIAS ANOCA trial add to previous findings in CorMicA and AID-ANGIO studies and indicate that routine ad hoc CFT is feasible and supports relevant medical therapy decisions that ultimately result in improved patient-reported outcomes.

### Strengths

These study findings are supported by several strengths. First, albeit challenged by COVID-19 and the global ACh shortage, we enrolled all-comer patients referred for ICA in the work-up of CCS, including those with suspected CAD on CCTA, from a variety of referral centres. As such, we could evaluate the safety, feasibility, and clinical value of ad hoc CFT in contemporary clinical practice. Similar to the CorMicA trial, we employed a practical CFT protocol without the need for withholding vasoactive medication, with lenience towards operator preference for the use of radial cocktail, which allowed for physiological interrogation of intermediate epicardial lesions prior to embarking on CFT. Following CorMicA, AID-ANGIO, and a previous study evaluating the impact of radial cocktail on CFT results, the ILIAS ANOCA trial documents the high diagnostic yield and clinical impact of such a practical approach to CFT, increasing the potential for its uptake in daily clinical practice.^4,9,18^ Moreover, this trial deepened our understanding of the stratified treatment of ANOCA endotypes in a non-expert setting. Most patients in the ILIAS ANOCA trial were referred for ICA from external outpatient clinics, which are not directly connected to the expert centres conducting ICA and CFT. Despite this, the information derived from the CFT and its accompanying medical therapy protocol demonstrated a significant treatment effect, even when most patients received treatment outside of specialized ANOCA clinics, mirroring real-world clinical practice. Finally, this study is the first to use Doppler-based physiological assessment for the implementation of ad hoc CFT, including HMR. Doppler-based assessment allows for the direct and continuous assessment of coronary flow throughout the CFT. As such, the occurrence of flow disturbances can be noticed early and directly during the infusion of ACh, increasing the safety of the procedure. In addition, Doppler-based flow assessment allows for the use of intracoronary adenosine for induction of coronary hyperaemia, which is notably quick and shortens procedure time compared with techniques that require infusion protocols.

### Limitations

Our study has some limitations that should be considered when interpreting the results. First, due to the nature of CFT and the need for assessment of patient-reported symptoms during the ACh provocation, it is impossible to conduct a fully blinded randomized CFT study protocol, which could potentially introduce bias. Nevertheless, the trained catheterization laboratory staff, clinical researchers, and interventional cardiologist ensured blinding from the CFT results. In addition, the results are based on patient-reported outcomes and, although possibly more valuable in ANOCA patients, are susceptible to bias. We defined ANOCA endotypes based on binary and pre-defined cut-offs for CFR and HMR and the occurrence of vasospasm. Albeit following clinical consensus for their diagnosis, specific responses to CFT not fitting with these formal criteria may still benefit from symptomatic treatment. Further analyses will focus on the impact of endotypes and responses to ACh testing, as well as additional information provided by endothelial function testing, on the observed treatment response.

## Conclusion

Routine CFT during the initial ICA for CCS was feasible, safe, and identified a coronary vasomotor disorder in 78% of ANOCA patients. Implementing a pragmatic CFT protocol combined with a disease-specific treatment protocol significantly improved disease-related quality of life and angina frequency in patients with ANOCA compared with standard care. These findings support the integration of CFT-guided care into routine clinical practice.

## Supplementary Material

ehaf580_Supplementary_Data

## Declarations

### Data Availability

The data underlying this article will be shared on reasonable request to the corresponding author.

### Pre-registered Clinical Trial Number

The ILIAS ANOCA trial is registered within the International Clinical Trials Registry Platform by the following identifier: NL-OMON20739.

## References

[1] Kunadian V, Chieffo A, Camici PG, Berry C, Escaned J, Maas AHEM, et al An EAPCI expert consensus document on ischaemia with non-obstructive coronary arteries in collaboration with European Society of Cardiology Working Group on Coronary Pathophysiology & Microcirculation endorsed by Coronary Vasomotor Disorders International Study Group. Eur Heart J 2020;41:3504–20. 10.1093/eurheartj/ehaa503 32626906 PMC7577516

[2] Vrints C, Andreotti F, Koskinas KC, Rossello X, Adamo M, Ainslie J, et al 2024 ESC Guidelines for the management of chronic coronary syndromes. Eur Heart J 2024;45:3415–537. 10.1093/eurheartj/ehae177 39210710

[3] Feenstra RGT, Boerhout CKM, Woudstra J, Vink CEM, Wittekoek ME, Waard D, et al Presence of coronary endothelial dysfunction, coronary vasospasm, and adenosine-mediated vasodilatory disorders in patients with ischemia and nonobstructive coronary arteries. Circ Cardiovasc Interv 2022;15:E012017. 10.1161/CIRCINTERVENTIONS.122.012017 35904014

[4] Jerónimo A, Paredes-Vázquez JG, Travieso A, Shabbir A, Jiménez-Quevedo P, Macaya-Ten F, et al Comprehensive diagnosis in chronic coronary syndromes combining angiography and intracoronary testing: the AID-ANGIO study. EuroIntervention 2025;21:35–45. 10.4244/EIJ-D-24-00499 PMC11684331 39773829

[5] Crooijmans C, Jansen TPJ, Meeder JG, Woudstra J, Meuwissen M, De Vos AMJ, et al Safety, feasibility, and diagnostic yield of invasive coronary function testing: Netherlands registry of invasive coronary vasomotor function testing. JAMA Cardiol 2025;10:384–90. 10.1001/jamacardio.2024.5670 39969865 PMC11840684

[6] Ford TJ, Yii E, Sidik N, Good R, Rocchiccioli P, McEntegart M, et al Ischemia and no obstructive coronary artery disease: prevalence and correlates of coronary vasomotion disorders. Circ Cardiovasc Interv 2019;12:e008126. 10.1161/CIRCINTERVENTIONS.119.008126 31833416 PMC6924940

[7] Sara JD, Widmer RJ, Matsuzawa Y, Lennon RJ, Lerman LO, Lerman A. Prevalence of coronary microvascular dysfunction among patients with chest pain and nonobstructive coronary artery disease. JACC Cardiov Interv 2015;8:1445–53. 10.1016/j.jcin.2015.06.017 26404197

[8] Lee BK, Lim HS, Fearon WF, Yong AS, Yamada R, Tanaka S, et al Invasive evaluation of patients with angina in the absence of obstructive coronary artery disease. Circulation 2015;131:1054–60. 10.1161/CIRCULATIONAHA.114.012636 25712205 PMC5295466

[9] Ford TJ, Stanley B, Good R, Rocchiccioli P, McEntegart M, Watkins S, et al Stratified medical therapy using invasive coronary function testing in angina: the CorMicA trial. J Am Coll Cardiol 2018;72:2841–55. 10.1016/j.jacc.2018.09.006 30266608

[10] Boerhout CKM, Namba HF, Liu T, Beijk MAM, Damman P, Meuwissen M, et al Rationale and design of the ILIAS ANOCA clinical trial: a blinded-arm controlled trial for routine ad-hoc coronary function testing. Am Heart J 2025;286:1–13. 10.1016/j.ahj.2025.03.004 40068714

[11] Seitz A, Feenstra R, Konst RE, Martínez Pereyra V, Beck S, Beijk M, et al Acetylcholine rechallenge: a first step toward tailored treatment in patients with coronary artery spasm. JACC Cardiovasc Interv 2022;15:65–75. 10.1016/j.jcin.2021.10.003 34991826

[12] Thomas M, Jones PG, Arnold SV, Spertus JA. Interpretation of the Seattle Angina Questionnaire as an outcome measure in clinical trials and clinical care: a review. JAMA Cardiol 2021;6:593–9. 10.1001/jamacardio.2020.7478 PMC8651216 33566062

[13] Herdman M, Gudex C, Lloyd A, Janssen M, Kind P, Parkin D, et al Development and preliminary testing of the new five-level version of EQ-5D (EQ-5D-5L). Qual Life Res 2011;20:1727–36. 10.1007/s11136-011-9903-x 21479777 PMC3220807

[14] Leone AM, Galante D, Viceré A, Marrone A, Verardi FM, Giuliana C, et al Functional coronary assessment in angina with intermediate coronary stenosis: the #FullPhysiology approach. Eur Heart J 2025;46:978–80. 10.1093/eurheartj/ehae926 39791532 PMC11887535

[15] Feenstra RGT, Seitz A, Boerhout CKM, Bukkems LH, Stegehuis VE, Teeuwisse PJI, et al Principles and pitfalls in coronary vasomotor function testing. EuroIntervention 2022;17:1271–80. 10.4244/EIJ-D-21-00402 34278990 PMC9725006

[16] Spertus JA, Jones PG, Maron DJ, O’Brien SM, Reynolds HR, Rosenberg Y, et al Health-status outcomes with invasive or conservative care in coronary disease. N Engl J Med 2020;382:1408–19. 10.1056/NEJMoa1916370 32227753 PMC7261489

[17] Zheng Y, Dou L, Fu Q, Li S. Responsiveness and minimal clinically important difference of EQ-5D-5L in patients with coronary heart disease after percutaneous coronary intervention: a longitudinal study. Front Cardiovasc Med 2023;10:1074969. 10.3389/fcvm.2023.1074969 36970361 PMC10034178

[18] Schäufele T, Menezes MN, Steinberg BS, Hubert A, Pereyra VM, Arndt H, et al Effects of radial artery spasm prophylaxis on intracoronary vasomotor responses during acetylcholine spasm provocation testing. Int J Cardiol 2024;419:132703. 10.1016/j.ijcard.2024.132703 39491593

