# Supplementary material for: Coronary function testing vs angiography alone to guide treatment of angina with non-obstructive coronary arteries: the ILIAS ANOCA trial
Source: Eur Heart J. 2025 Aug 12;46(42):4396–406. doi: 10.1093/eurheartj/ehaf580 (PMC12596479; doi:10.1093/eurheartj/ehaf580)

# Supplementary figure 1

1. Treatment protocol for endotype “abnormal vasodilatation”
2. Treatment protocol for endotype “abnormal vasoconstriction”
3. Treatment protocol for endotype “mixed abnormal vasodilatation & abnormal vasoconstriction”
4.
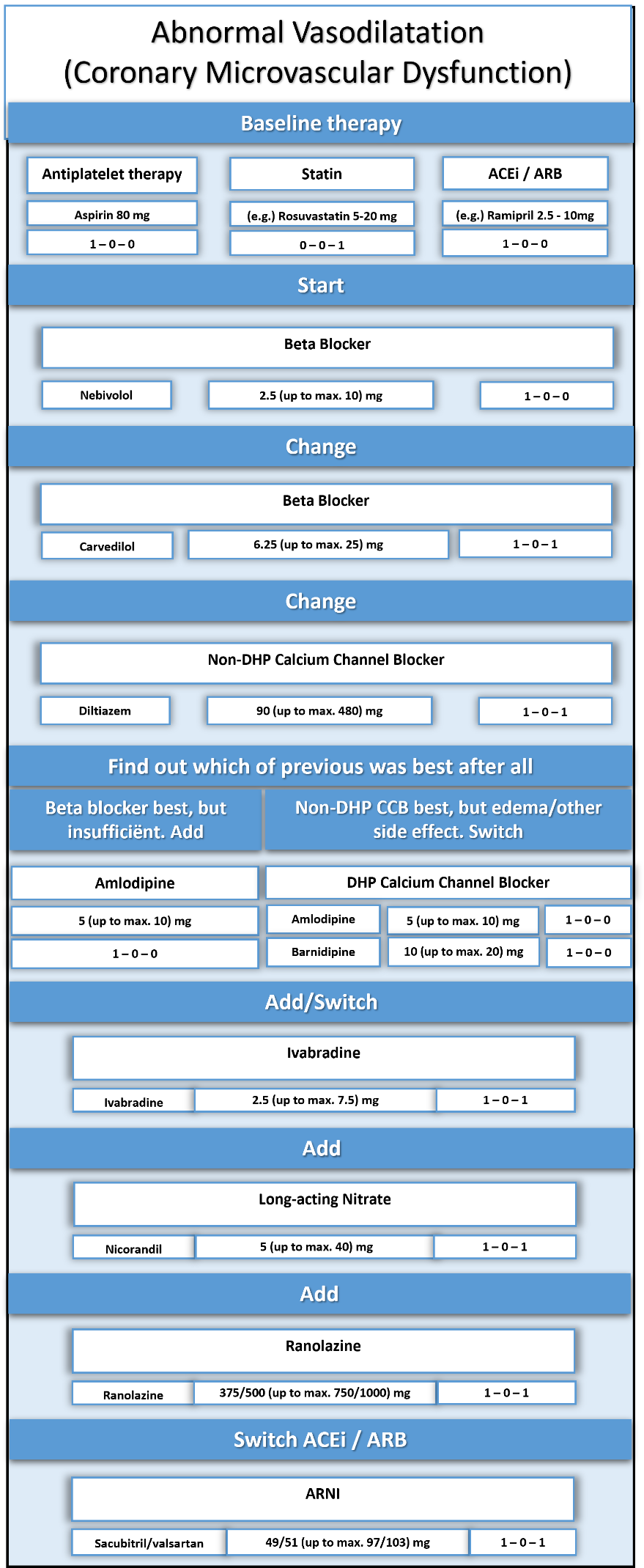

5.
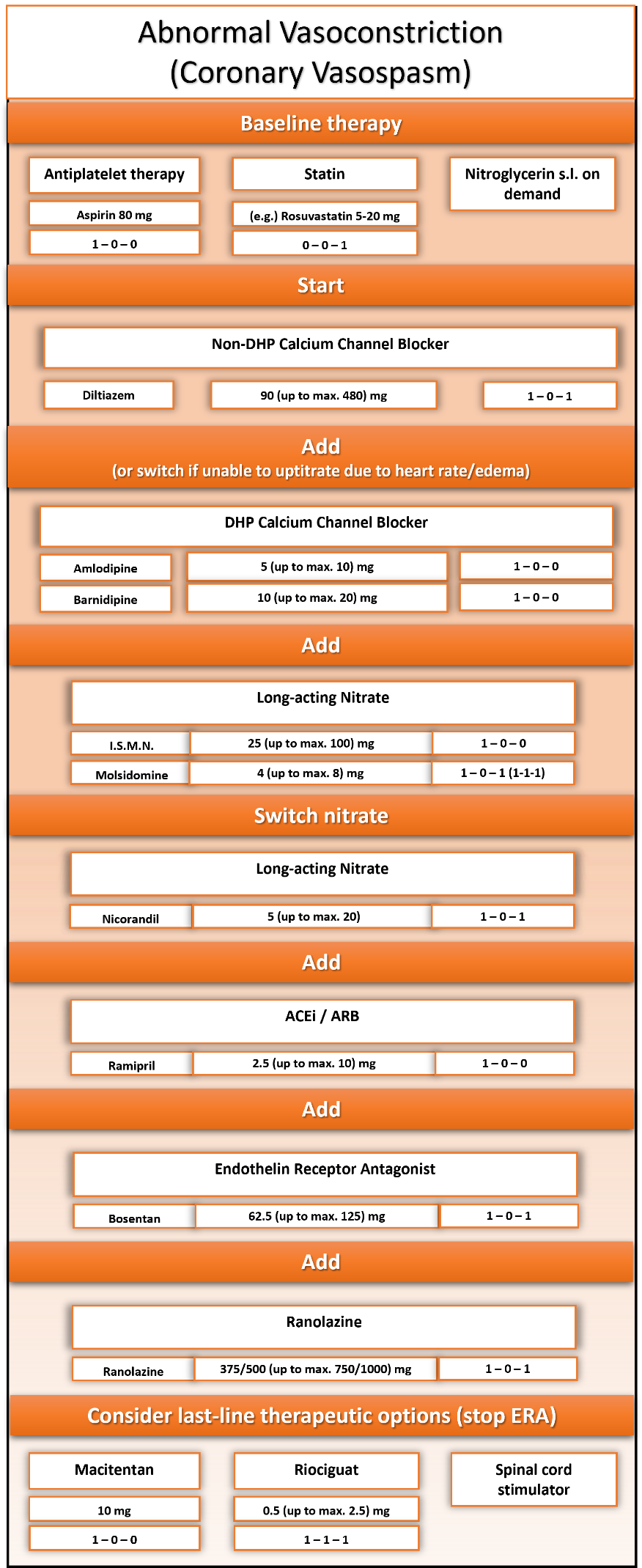

6.
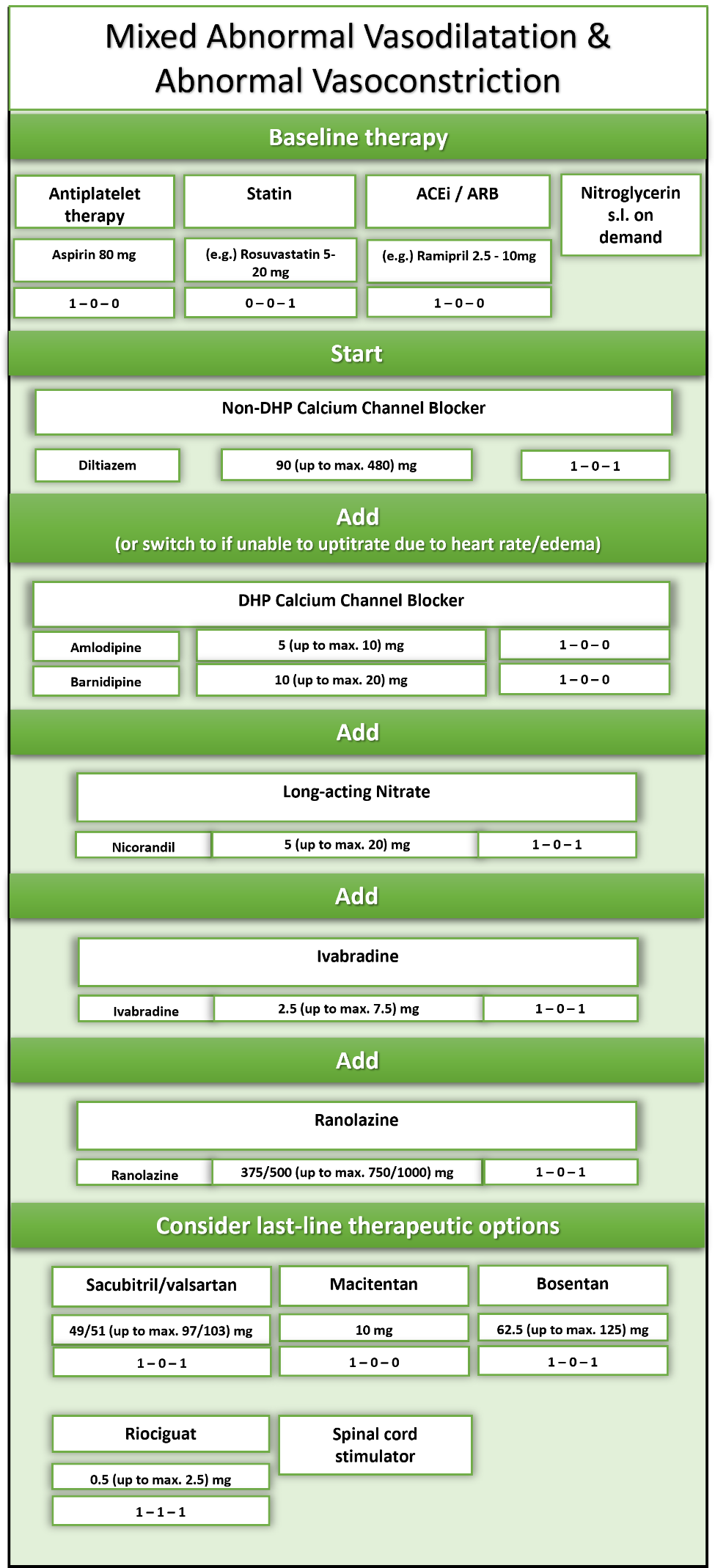

Supplement: ehaf580_Supplementary_Data [file ehaf580_supplementary_data.docx]
